# Model-Based Nonlinear Feedback Controllers for Pressure Control of Soft Pneumatic Actuators Using On/Off Valves

**DOI:** 10.3389/frobt.2022.818187

**Published:** 2022-03-16

**Authors:** Matheus S. Xavier, Andrew J. Fleming, Yuen K. Yong

**Affiliations:** Precision Mechatronics Lab, The University of Newcastle, Callaghan, NSW, Australia

**Keywords:** soft pneumatic actuators, soft robotics, pneumatic systems, nonlinear control, SDRE, soft actuator

## Abstract

This article describes the application and comparison of three nonlinear feedback controllers for low-level control of soft actuators driven by a pressure source and single high-speed on/off solenoid valve. First, a mathematical model of the pneumatic system is established and the limitations of the open-loop system are evaluated. Next, a model of the pneumatic system is developed using Simscape Fluids to evaluate the performance of various control strategies. In this article, State-Dependent Riccati Equation control, sliding mode control, and feedback linearization are considered. To improve robustness to model uncertainties, the sliding mode and feedback linearization control strategies are augmented with integral action. The model of the pneumatic system is also used to develop a feedforward component, which is added to a PI controller with anti-windup. The simulation and experimental results demonstrate the effectiveness of the proposed controllers for pressure tracking.

## 1 Introduction

Soft robotics is a rapidly growing field in which the robots are elastically deformable (Rus and Tolley, 2015; Bao et al., 2018; Xu and Wang, 2021). Soft robots can be actuated using dieletric elastomers, shape-memory alloys, magnetic actuation, photo-responsive materials, tendon-driven approaches, or fluid power (El-Atab et al., 2020; Yang et al., 2020). Pneumatic or hydraulic soft robots are used in applications such as minimally invasive surgery (Runciman et al., 2019), rehabilitation (Wehner et al., 2013), elderly assistance (Ansari et al., 2017), safe human–robot interaction (De Greef et al., 2009), and handling of fragile materials (Shintake et al., 2018). Fluid-driven soft robots offer high dexterity and safety, large deformation, good power-to-weight ratio, and low manufacturing cost (El-Atab et al., 2020; Tawk and Alici, 2021). Soft robots are fabricated from soft actuators, including single-, double-, and multi-chambered actuators, fiber-reinforced actuators, and omnidirectional actuators (Gorissen et al., 2017; Xavier et al., 2021a).

Soft pneumatic actuators are usually controlled with proportional or on/off solenoid valves (Skorina et al., 2017; Booth et al., 2018; Wang et al., 2019a; Young et al., 2021). The most popular pneumatic control architecture for soft robotics is the fluidic control board, an open source hardware platform available from the Soft Robotics Toolkit (Soft robotics toolkit, 2019) that was originally employed in the experimental platform of Galloway et al. (2013), Polygerinos et al. (2013), and Polygerinos et al. (2015) and has since then inspired many pneumatic control systems (Onal and Rus, 2013; Luo et al., 2014a; Luo et al., 2014b). The board consists mainly of a diaphragm pump, a set of solenoid valves, and pressure sensors for feedback control. MOSFETs allow the use of Pulse-Width Modulation (PWM) to control the pressure of fluid passing through the valves. Pressure can also be controlled using pressure regulators; however, this allows only one pressure in the whole system. Recently, a number of other open-source pneumatic drivers have also been proposed such as FlowIO (Shtarbanov, 2021), Pneuduino (Ou et al., 2016), and PneuSoRD (Young et al., 2021).

The literature describes various model-based nonlinear control strategies for pneumatic cylinders and hydraulic systems (Rahmat et al., 2011; Weist et al., 2011; Saravanakumar et al., 2017), such as sliding modes (Paul et al., 1994; Richer and Hurmuzlu, 2000; Barth et al., 2002; Nguyen et al., 2007), State-Dependent Riccati Equation (SDRE) (Weickgenannt et al., 2010; Strano and Terzo, 2015; Strano and Terzo, 2016), feedback linearization (Lee et al., 2002; Ke et al., 2007), and adaptive (Tsai and Huang, 2008; Zhihong Rao and Bone, 2008) and fuzzy (Shih and Ma, 1998; Chillari et al., 2001) controllers. In contrast to their rigid counterparts, model-based dynamic controllers for the more recent soft pneumatic actuators are still in their nascent stage (Skorina et al., 2017; Wang et al., 2019a). For pneumatic-driven soft robots, the nonlinearities arising from hyperelastic materials, complex geometries, and the compressibility of air hinder the development of accurate mathematical models. Analytical models for soft actuators have been developed using the piecewise constant curvature approach (Webster and Jones, 2010; Marchese et al., 2014a), the Lagrangian approach (Wang et al., 2019b; Cao et al., 2021a), the Euler–Bernoulli principle (Gorissen et al., 2011; Polygerinos et al., 2015; Xiao et al., 2021), the Castigliano method (Drotman et al., 2017; Drotman et al., 2019), the theory of Cosserat rods (Bartholdt et al., 2021; Berthold et al., 2021), and data-driven approaches (Elgeneidy et al., 2018; Mohamed et al., 2020). Alternatively, a modeling framework for pneumatic systems can be developed using an energy-based approach to derive lumped parameter models for fluid circuit components (Watton, 1989; De Silva, 2004; Karnopp et al., 2012). In particular, pneumatic sources act as current sources, fluidic tubing and channels act as impedances, and fluidic chambers act as capacitances (Onal and Rus, 2013; Marchese et al., 2014a; Xavier et al., 2020). Relying on the electrical circuit equivalence of pneumatic systems, the dynamic behavior of a bending soft actuator can be approximated as a lumped second-order dynamic equation (Onal and Rus, 2013; Skorina et al., 2015).

Using this second-order equation, sliding mode controllers are developed in the works of Skorina et al. (2015), Luo et al. (2017), and Khan and Li (2020) to control the bending angle of soft actuators governed by high-speed on/off solenoid valves. A sliding mode controller with a static mapping function to create a feedforward augmented sliding mode controller is proposed by Skorina et al. (2015), which improved tracking for dynamic trajectories under a payload. A model reference adaptive controller augmented by a feedforward inverse dynamic controller is used by Skorina et al. (2017) to demonstrate the versatility of the proposed control approach. Alternatively, a purely data-driven approach can be used to control the bending angle of soft actuators (Elgeneidy et al., 2018). An observer-based adaptive sliding mode controller using a dynamic model on the basis of the Euler–Lagrange method is proposed by Cao et al. (2021b) to estimate the velocity information and track desired bending angle references.

Experimentally tuned PID and on-off controllers have also been extensively used in fluid-powered soft robots (Khan et al., 2020; Xavier et al., 2021b), such as snake-like (Onal and Rus, 2013), worm-like (Calderón et al., 2016), soft-bodied fish (Marchese et al., 2014b), and manta ray (Suzumori et al., 2007) robots. Automatic tuning of ordinary, piecewise, and fuzzy PI controllers using a heuristic-based coordinate descent algorithm is proposed by Khan et al. (2020), which was shown to generally produce better results than manually tuned parameters. The sliding mode controller in the work of Ibrahim et al. (2019) outperformed the PID controller in the simulation results; however, the PID controller performed best in experimental work, at the expense of higher overshoot and lower robustness to external forces. Conversely, the sliding mode controller with a PID sliding surface in the work of Khan and Li (2020) dampens vibration on deactuation in comparison to an experimentally tuned PID controller.

The articles described above have presented controllers for bending angle or extension motions, i.e., high-level control (George Thuruthel et al., 2018). However, few works have considered the impact of the pneumatic system on the soft actuator performance and developed low-level control (pressure control) strategies. Regardless of the soft actuator design, the pneumatic system critically affects the pressure dynamics of soft actuators (Xavier et al., 2020; Joshi and Paik, 2021). While the actuation mode, force, and displacement are governed by the actuator design and loading conditions, the actuation speed is largely determined by the pressure and flow dynamics of the soft pneumatic actuator (Joshi et al., 2021). Therefore, pressure control plays a major role in the overall performance of soft robots (Skorina et al., 2017; George Thuruthel et al., 2018). In the work of Wang et al. (2019a), a pneumatic model was used to control the bending angle of a pneumatic network actuator using a robust backstepping controller with two-way, two-position on/off valves. Sliding mode controllers are proposed by Ibrahim et al. (2019; 2021) to control the pressure of a soft actuator using proportional valves. In the work of Chen et al. (2020), a pneumatic model is included to control the bending angle of a fiber-reinforced actuator using two three-way, two-position on/off valves with a backstepping-based adaptive robust controller and sliding mode controller. In the work of Falkenhahn et al. (2016), feedback linearization is proposed to control the motion of a bellow-shaped continuum manipulator with proportional valves. Sliding mode controllers are also used by Jouppila et al. (2014) for position control of a pneumatic muscle in a comparative study between three approaches using on/off valves and traditional servo valves. Finally, cascade control structures have also been proposed where the faster inner layer performs pressure control and outer layer is responsible for open-loop angle control (Yi et al., 2018; Zhou et al., 2019).

### 1.1 Contributions

**FIGURE 1:**
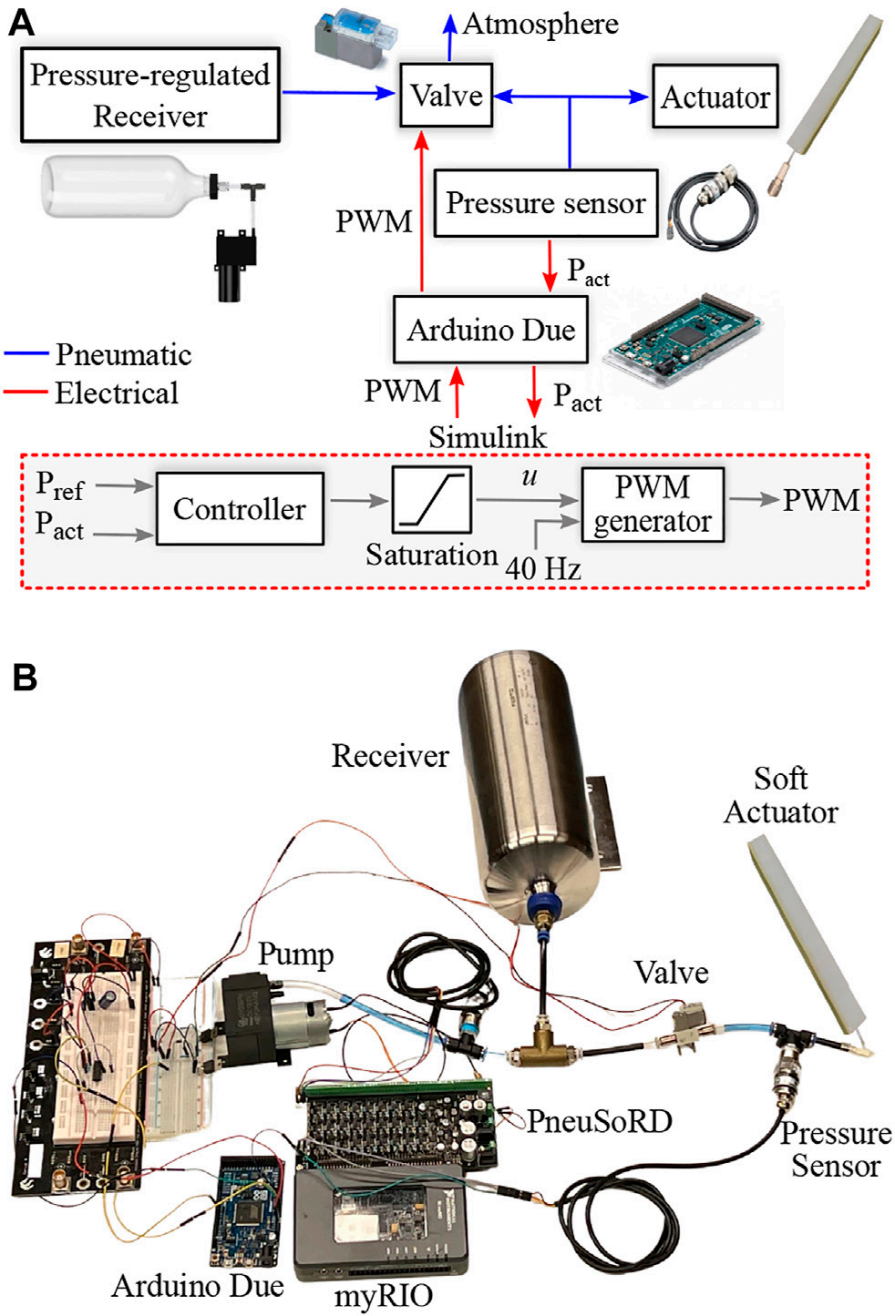
Pneumatic system with 3/2 valve and pressure-regulated receiver. The pressure of the soft actuator is regulated by controlling the duty cycle of the valve with a 40-Hz PWM signal. The dotted red box illustrates the implementation of the control strategies in Simulink. **(A)** High-level description. **(B)** Physical system.

In this article, analytical and simulations models are developed for the pressure dynamics of soft pneumatic actuators governed by pneumatic systems with three-way, two-position on/off valves and a pressure-regulated receiver, as shown in Figure 1. On the basis of the analytical model, three nonlinear feedback controllers are derived for low-level control of soft actuators: SDRE, sliding mode, and feedback linearization. The tracking performance and robustness of these controllers is enhanced by augmenting the sliding mode and feedback linearization controllers with integral action. The mathematical model is also used to determine a feedforward component that is augmented to a PI controller with anti-windup. The control strategies are evaluated on a simulation model developed in Simscape Fluids and also experimentally on a bending soft pneumatic actuator using an Arduino Due and Simulink. The performance of the controllers is evaluated using metrics for the tracking performance and control effort. The simulation and experimental results demonstrate the effectiveness of the proposed nonlinear controllers for pressure tracking.

The remainder of this article is organized as follows. Section 2 describes the air dynamics model for the soft actuator, and Section 3 presents the parameter selection for the pneumatic system. Section 4 describes the model-based nonlinear controllers used in this study. Section 5 presents simulation results for the control strategies based on a model developed within Simscape Fluids. In Section 6, experimental results of the closed-loop tracking performances are evaluated and compared to the simulations. Finally, Section 7 discusses the conclusions of this work.

## 2 Modeling

The pneumatic system in this work employs a single three-way, two-position on/off valve, a diaphragm pump, and pressure sensors for feedback control, as shown in Figure 1. An air receiver (reservoir) is employed to improve response speed and efficiency while minimizing the peak pump flow rate. The inlet port of the valve is connected to the receiver; the outlet port is connected to the soft actuator. Pressure control in the actuator is performed by controlling the duty cycle of the PWM wave into the valve.

### 2.1 Fundamental Equations

Two fundamental equations are used to model the air pressure dynamics of the pneumatic system. The polytropic gas law is used to model the actuator pressurization, and the valve model ANSI/(NFPA)T3.21.3 is used to characterize the flow rate during charging and discharging.

1) Polytropic gas law (equation of state)

$$PV^{\gamma }=constant$$ (1)

where P is the pressure, V is the volume, and γ is the polytropic index. For an isothermal process, γ=1 , whereas, for an isentropic process (adiabatic and reversible), $\gamma =k=c_{p}/c_{v}=1.4$ . The pressure and volume in the equation above may have various different units. In this work, P has absolute bar units to comply with the valve model and V is in liters. However, note that the pressure is measured and presented in relative kilopascals for the simulation and experimental results; hence, appropriate unit conversions are adopted as required. Differentiating both sides,

$$\gamma PV^{\gamma -1}\frac{dV}{dt}+V^{\gamma }\frac{dP}{dt}=0\Rightarrow Q=\frac{V}{\gamma P}\frac{dP}{dt}$$ (2)

where $Q=-\frac{dV}{dt}$ is the volume flow rate into the actuator.

2) Valve model ANSI/(NFPA)T3.21.3

Volumetric flow rate Q (L/s) through a valve is given by

$$Q=\frac{114.5uC_{v}\sqrt{P_{low}\Delta P}}{\sqrt{T_{high}}}$$ (3)

where $\Delta P=P_{high}-P_{low}$ , $P_{high}$ and $P_{low}$ are the absolute upstream and downstream pressures (bar), u is the duty cycle of the PWM signal applied to the valve, $C_{v}$ is the flow coefficient, and $T_{high}$ is the upstream temperature (kelvin).

### 2.2 Pressure Dynamics of Actuator

The net flow into the soft actuator is given by $Q=Q_{c}-Q_{d}$ , where $Q_{c}$ and $Q_{d}$ are the flow rates for the charging and discharging valves. Then, in Eq. 2,

$$\frac{dP_{A}}{dt}=\frac{\gamma P_{A}}{V_{A}}\left(Q_{c}-Q_{d}\right)$$ (4)

where $P_{A}$ (absolute bar) and $V_{A}$ (L) are the pressure and volume of the actuator. From Eq. 3,

$$Q_{c}=\frac{114.5u_{c}C_{v}\sqrt{P_{A}\left(P_{R}-P_{A}\right)}}{\sqrt{T_{R}}}$$ (5)

$$Q_{d}=\frac{114.5u_{d}C_{v}\sqrt{P_{atm}\left(P_{A}-P_{atm}\right)}}{\sqrt{T_{A}}}$$ (6)

where $u_{c}$ and $u_{d}$ are the duty cycles for the charging and discharging valves, respectively; $P_{R}$ is the receiver pressure (absolute bar); and $T_{R}$ and $T_{A}$ are receiver and actuator temperatures (kelvin), respectively. Here, $T_{R}=T_{A}=T=293.15$ K.

Inserting Eqs. 4, 5 into Eq. 4 and defining $\alpha =\frac{114.5\gamma C_{v}}{V_{A}\sqrt{T}}$ yields

$$\frac{dP_{A}}{dt}=\alpha P_{A}\left(u_{c}\sqrt{P_{A}\left(P_{R}-P_{A}\right)}-u_{d}\sqrt{P_{atm}\left(P_{A}-P_{atm}\right)}\right)$$ (7)

For pneumatic systems with a 3/2 valve, a single PWM signal is used; hence, $u_{c}=u$ and $u_{d}=\left(1-u\right)$ . Set $x=P_{A}$ , $c_{1}=P_{R}$ , and $c_{2}=P_{atm}$ in Eq. 7; then, the actuator model takes the form

$$\dot{x}=f\left(x\right)+g\left(x\right)u$$ (8)

$$f\left(x\right)=-\alpha x\sqrt{c_{2}\left(x-c_{2}\right)}$$ (9)

$$g\left(x\right)=\alpha x\left(\sqrt{x\left(c_{1}-x\right)}+\sqrt{c_{2}\left(x-c_{2}\right)}\right)$$ (10)

## 3 Open-Loop System Design

Prior to evaluating the performance of nonlinear controllers, the open-loop system response is evaluated to determine the physical limitations of the pneumatic system and select the required pneumatic components given soft actuator specifications and desired pressure response characteristics. A parameter selection procedure is proposed by (Xavier et al. (2021b) using the ISO6358 standard for the valves, which can be converted to ANSI/(NFPA) T3.21.3 after valve selection. Following the procedure in the work of Xavier et al. (2021b).

1) Define actuator characteristics and requirements:

• Desired pressure $P_{A}$ and volume $V_{A}$ of the actuator: $P_{A}=$ 60 kPa (161.325 kPa absolute), $V_{A}=$ 30 ml.

• Desired rise time $T_{rise}$ of the response: $T_{rise}=$ 0.25 s.

• Maximum number of actuation cycles per minute N : N= 30 cycles/min.
2) Select receiver parameters:

• $V_{R}>10\times V_{A}$ : $V_{R}=$ 2 L.

• $P_{R}>1.2\times P_{A}$ and $P_{R}\in \left\{100,150,200\right\}\text{kPa}$ : $P_{R}=$ 100 kPa.
3) Select valve sonic conductance C :
  •Use Figure 5 in the work of Xavier et al. (2021b) to determine the normalized conductance $\bar{C}$ for desired $P_{A}$ and $T_{rise}$ : $\bar{C}=$ 1.04.

• For the corresponding $\bar{C}$ , select $C\geq \bar{C}\times V_{A}$ :

C≥ 0.031 L/(s ⋅ bar).
4) Select valve configuration:

• three-way, two-position V114 (SMC) with C=0.037 L/(s ⋅ bar). Therefore, $C_{v}=0.008$ .
5) Select air pump flow in liters per minute (LPM):
  •From Eq. 15, $Q_{Pump}\left(P_{R}\right)\geq Q_{req,3way}$ = 0.6 + 1.3 = 1.9 LPM. Hence, the KYK50BPM pump is selected.
  From Equation 4, the volume of air $V_{air}$ (L) consumed during one actuation cycle from atmospheric pressure $P_{atm}$ to the desired pressure $P_{A}$ is

$$V_{air}=\frac{V_{A}}{\gamma }\ln\left(\frac{P_{A}}{P_{atm}}\right)$$ (11)

Consequently, for N actuation cycles per minute, the required flow $Q_{req}$ (LPM) from the air pump to keep the receiver at constant pressure is

$$Q_{req}=\frac{N\times V_{A}}{\gamma }\ln\left(\frac{P_{A}}{P_{atm}}\right)$$ (12)

For three-way valves, an additional volume of air is consumed during regulation due to frequent switching and release of air to the atmosphere. The additional flow in liters per second is

$$Q_{add}=\frac{114.5u_{ff}C_{v}\sqrt{P_{A}\left(P_{R}-P_{A}\right)}}{\sqrt{T}}$$ (13)

where $u_{ff}$ is the required duty cycle for a desired pressure. This is obtained by setting $\dot{x}=0$ in Eq. 8, which results in

$$u_{ff}=\frac{\sqrt{c_{2}\left(x_{d}-c_{2}\right)}}{\sqrt{x_{d}\left(c_{1}-x_{d}\right)}+\sqrt{c_{2}\left(x_{d}-c_{2}\right)}}$$ (14)

Therefore, the total required flow for three-way valves $Q_{req,3way}$ is

$$Q_{req,3way}=Q_{req}+60\times Q_{add}\;\left[\text{in LPM}\right]$$ (15)

Therefore, the setup investigated here employs the diaphragm pump KYK50BPM, a V114 on/off valve, a 2 L receiver, two pressure sensors (SEN0257, DFRobot) and tubing between each of these elements. The pneumatic soft robotics driver (PneuSoRD) proposed by Young et al. (2021) is used with myRIO to control the pressure of the receiver and minimize sensor noise due to switching of the valve and motor. The inlet port of the valve is connected to the receiver, the outlet port is connected to the soft actuator and the exhaust port is open to atmosphere. The PWM output for the motor is connected to PneuSoRD (Young et al., 2021) and the PWM outputs for the valves are each connected to a BJT transistor (TIP31A) through a 330 Ω resistor. A flyback diode (1n5817) is added to dissipate energy stored in the solenoid during turn-off.

## 4 Controller Design

In this section, the three nonlinear controllers are described, in addition to the PI controller with feedforward and anti-windup. SDRE control provides a systematic and effective algorithm for designing nonlinear controllers by allowing nonlinearities in the system states while additionally offering great design flexibility. Sliding mode controllers provide a systematic approach for robust control and have found extensive application in pneumatic systems because switched control laws can provide high performance for systems employing PWM. The feedback linearization approach has a straightforward implementation for single-input, single-output nonlinear systems in normal form (Eq. 8) and allows for the development of various state-space control techniques available for linear systems, such as pole placement and linear quadratic regulator design.

### 4.1 State-Dependent Riccati Equation Control

The general infinite-horizon nonlinear regulator problem is defined by the minimization of the following performance criterion:

$$J=\int_{0}^{\infty }x^{T}\left(t\right)Q\left(x\right)x\left(t\right)+u^{T}\left(t\right)R\left(x\right)u\left(t\right)\;dt$$ (16)

with respect to the state x and the control u subject to the nonlinear differential constraint Eq. 8, where Q(x)≥0 and R(x)>0 for all x , and $f\left(x\right),g\left(x\right),R\left(x\right),Q\left(x\right)\in C^{k}$ , k≥1 . The controller parameters can be tuned by recalling that Q penalizes the tracking error and R penalizes control authority. If the elements of Q are large compared to the elements of R , then the states remain small, whereas large values for R guarantee small control inputs.

The SDRE design proceeds as follows (Mracek and Cloutier, 1998; Çimen, 2010; Nekoo, 2019).

1) Use direct parameterization to bring the nonlinear dynamics to the State-Dependent Coefficient form, i.e.,

$$\dot{x}=A\left(x\right)x+B\left(x\right)u$$ (17)

where

f(x)=A(x)x , B(x)=g(x) (18)

2) Solve the SDRE

$$P\left(x\right)A\left(x\right)+A^{T}\left(x\right)P\left(x\right)-P\left(x\right)B\left(x\right)R^{-1}\left(x\right)B^{T}\left(x\right)P\left(x\right)+Q\left(x\right)=0$$ (19)

3) Construct the nonlinear feedback controller

$$u=-R^{-1}\left(x\right)B^{T}\left(x\right)P\left(x\right)x$$ (20)

For regulation to non-zero set points, the following error variables are defined: $\tilde{x}=x-x_{d}$ and $\tilde{u}=u-u_{d}$ , where $x_{d}$ and $u_{d}$ are the desired (or reference) values. The control objective becomes the minimization of the error, i.e., $\tilde{x}\to 0$ as t→∞ , which implies $x\to x_{d}$ as t→∞ . The following error system is obtained (refer to Appendix for derivation),

$$\dot{\tilde{x}}=A\left(\tilde{x}\right)\tilde{x}+B\left(\tilde{x}\right)\tilde{u}$$ (21)

where

$A\left(\tilde{x}\right)=-\alpha a_{1}+\alpha a_{2}u_{d}+\alpha a_{1}u_{d}+a_{3}/\tilde{x}$

$B\left(\tilde{x}\right)=\alpha x_{d}a_{2}+\alpha \tilde{x}a_{2}+\alpha x_{d}a_{1}+\alpha \tilde{x}a_{1}$

$a_{1}=\sqrt{c_{2}\left(x_{d}+\tilde{x}-c_{2}\right)}$ , $a_{2}=\sqrt{\left(x_{d}+\tilde{x}\right)\left(c_{1}-x_{d}-\tilde{x}\right)}$

$a_{3}=-\alpha x_{d}a_{1}+\alpha x_{d}a_{2}u_{d}+\alpha x_{d}a_{1}u_{d}+\alpha x_{d}a_{1d}-\alpha x_{d}a_{2d}u_{d}-\alpha x_{d}a_{1d}u_{d}$

$a_{1d}=\sqrt{c_{2}\left(x_{d}-c_{2}\right)}$ , $a_{2d}=\sqrt{x_{d}\left(c_{1}-x_{d}\right)}$

From step (2), the SDRE is

$$2P\left(\tilde{x}\right)A\left(\tilde{x}\right)-\frac{P^{2}\left(\tilde{x}\right)B^{2}\left(\tilde{x}\right)}{R\left(\tilde{x}\right)}+Q\left(\tilde{x}\right)=0$$ (22)

whose positive-definite solution is

$$P\left(\tilde{x}\right)=\frac{R\left(\tilde{x}\right)}{B^{2}\left(\tilde{x}\right)}\left(A\left(\tilde{x}\right)+\sqrt{A^{2}\left(\tilde{x}\right)+\frac{B^{2}\left(\tilde{x}\right)Q\left(\tilde{x}\right)}{R\left(\tilde{x}\right)}}\right)$$ (23)

From step (3), the control law for the error system is

$$\tilde{u}=-\frac{1}{B\left(\tilde{x}\right)}\left(A\left(\tilde{x}\right)+\sqrt{A^{2}\left(\tilde{x}\right)+\frac{B^{2}\left(\tilde{x}\right)Q\left(\tilde{x}\right)}{R\left(\tilde{x}\right)}}\right)\tilde{x}$$ (24)

Therefore, the overall control is $u=u_{d}+\tilde{u}$ , which has a feedback component $\tilde{u}$ and a feedforward component $u_{d}=u_{ff}$ given by Eq. 14. For this scalar system, the SDRE nonlinear feedback solution and its associated state $\tilde{x}$ and co-state $\lambda =P\left(\tilde{x}\right)\tilde{x}$ satisfy the conditions for optimality of the nonlinear regulator problem, i.e., $H_{u}=0$ and $\dot{\lambda }=-H_{x}$ , where H is the Hamiltonian of the system (Mracek and Cloutier, 1998; Naidu, 2002). Because the performance index is convex and the differential constraint is linear in u and because u is a scalar, there exists only one solution which is, therefore, globally asymptotically stable and globally optimal (Çimen, 2012). In addition, because the SDRE can be solved analytically, $\lim_{\left|\tilde{x}\right|\to 0}A\left(\tilde{x}\right)$ does not have to be finite as long as $\lim_{\left|\tilde{x}\right|\to 0}A\left(\tilde{x}\right)\tilde{x}$ is finite. For further details on the proof that these necessary conditions are satisfied and the derivation of the SDRE control law, readers are referred to the works of Cloutier et al. (1996) and Mracek and Cloutier (1998). Note that this exact solution greatly simplifies the implementation of the control law because it does not require the Riccati equation to be solved in each time step. Exact solutions are also proposed by Nekoo and Geranmehr (2013) and Rafee Nekoo (2013), where an online control update formulation is also discussed for more complex systems.

### 4.2 Integral Augmented Sliding Mode Controller

The conventional sliding surface for sliding mode control (SMC) is given by Slotine and Li (1991)

$$s\left(t\right)={\left(\frac{d}{dt}+\lambda \right)}^{n-1}\tilde{x}\left(t\right)$$ (25)

where n is the order of the system and λ is a strictly positive constant. The problem of tracking $x=x_{d}$ is equivalent to that of remaining on the surface s(t) for all t>0 ; thus, the problem can be reduced to that of keeping the scalar quantity s at zero. The dynamics, while in sliding mode, is given by $\dot{s}=0$ , from which the equivalent control $\hat{u}$ is obtained. To satisfy the sliding condition and ensure that the system trajectories remain on the surface s(t)=0 despite uncertainty on the dynamics f , a discontinuous control term $u_{s}$ across s(t)=0 is added to $\hat{u}$ : $u=\hat{u}-k_{s}\mathrm{sgn}\left(s\right)$ , where the constant $k_{s}$ increases with the extent of parametric uncertainty (Slotine and Li, 1991). The two main uncertain parameters are the soft actuator volume, which increases during pressurization, and the receiver pressure, which oscillates due to rapid bursts of flow into the actuator. In general, larger $k_{s}$ results in faster rise time but increases chattering in the soft actuator response. Therefore, in practice, the $k_{s}$ values are increased until satisfactory transient performance is achieved while maintaining reasonable control chattering and minimizing control saturation.

The discontinuous control law can be smoothed to achieve a trade-off between chattering and the magnitude of the tracking error. This is achieved by introducing a thin boundary layer around the sliding surface. Hence, SMC law is

$$u=\hat{u}-k_{s}\text{sat}\left(\frac{s}{\Phi }\right)=\hat{u}-k_{s}\left\{\begin{array}{cc} \frac{s}{\Phi },\; & \left|\frac{s}{\Phi }\right|\leq 1\\ \mathrm{sgn}\left(\frac{s}{\Phi }\right),\; & \left|\frac{s}{\Phi }\right|>1 \end{array}\right.$$ (26)

where Φ is the boundary layer thickness.

The tracking accuracy can be improved by introducing integral action into the sliding surface (Eker and Akınal, 2008):

$$s\left(t\right)={\left(\frac{d}{dt}+\lambda \right)}^{n-1}\tilde{x}\left(t\right)+k_{i}\int_{t_{0}}^{t}\tilde{x}\left(\tau \right)\text{d}\tau$$ (27)

where $k_{i}$ is the integral gain.

A necessary condition for the output trajectory to remain on the sliding surface is

$$\dot{s}=0\Rightarrow \dot{\tilde{x}}+k_{i}\tilde{x}=0$$ (28)

Choosing $k_{i}>0$ , the characteristic polynomial $s+k_{i}=0$ is strictly Hurwitz; therefore, $\tilde{x}\to 0$ as t→∞ and the closed-loop system is globally asymptotically stable. From Eq. 28, the equivalent control is

$$\hat{u}=\frac{1}{g\left(x\right)}\left({\dot{x}}_{d}-f\left(x\right)-k_{i}\left(x-x_{d}\right)\right)$$ (29)

Therefore, the overall SMC is

$$u=\frac{1}{g\left(x\right)}\left({\dot{x}}_{d}-f\left(x\right)-k_{i}\left(x-x_{d}\right)\right)-k_{s}\text{sat}\left(\frac{s}{\Phi }\right)$$ (30)

with $s=\tilde{x}\left(t\right)+k_{i}\int_{t_{0}}^{t}\tilde{x}\left(\tau \right)\text{d}\tau$ , f(x) defined in Eq. 9 and g(x) in Eq. 10.

### 4.3 Feedback Linearization With Integral Action

Here, the pressure of the actuator is defined as the output of the system, i.e., y=h(x)=x . Because $\frac{\partial h}{\partial x}\neq 0$ , a linear input–output relationship for the system defined in Eq. 8 can be obtained with the control law (Marquez, 2003)

$$u=\frac{1}{L_{g}h\left(x\right)}\left(-L_{f}h\left(x\right)+v\right)$$ (31)

where $L_{f}h\left(x\right)=\frac{\partial h}{\partial x}f\left(x\right)$ is the Lie derivative of h with respect to f and $L_{g}h\left(x\right)=\frac{\partial h}{\partial x}g\left(x\right)$ is the Lie derivative of h with respect to g . Because $\frac{\partial h}{\partial x}=1$ , the control law becomes

$$u=\frac{1}{g\left(x\right)}\left(-f\left(x\right)+v\right)$$ (32)

with f(x) defined in Eq. 9 and g(x) in Eq. 10. This control law renders the linear differential equation

$$\dot{x}=v$$ (33)

To compensate for model uncertainties and improve reference tracking, integral action is introduced by augmenting the system with a state that integrates the tracking error $\dot{\sigma }=x-x_{d}$ . Hence, the state-space model is given by

$$\left[\begin{array}{c} \dot{\tilde{x}}\\ \dot{\sigma } \end{array}\right]=\left[\begin{array}{cc} 0 & 0\\ 1 & 0 \end{array}\right]\left[\begin{array}{c} \tilde{x}\\ \sigma \end{array}\right]+\left[\begin{array}{c} 1\\ 0 \end{array}\right]\left(v-{\dot{x}}_{d}\right)$$ (34)

Defining $w=v-{\dot{x}}_{d}$ , the gains k and $k_{i}$ in the control law $w=-k\tilde{x}-k_{i}\sigma$ can be obtained with standard controller design techniques for linear systems such as pole placement. In practice, the gain k is increased until no significant changes are observed in the rise time of the soft actuator response and $k_{i}$ is kept at low values because large $k_{i}$ gains were observed to make the response slower to initial set point changes. Therefore, the linear control component is

$$v=-k\tilde{x}\left(t\right)-k_{i}\int_{t_{0}}^{t}\tilde{x}\left(\tau \right)d\tau +{\dot{x}}_{d}\left(t\right)$$ (35)

Finally, the overall control is obtained by introducing Eq. 35 into Eq. 32.

### 4.4 PI Controller With Feedforward and Anti-windup

The nonlinear controllers discussed above are compared to a PI controller augmented with feedforward. The inclusion of feedforward improves reference tracking and reduces the control effort from the feedback component, which is responsible to compensate for model uncertainty, i.e., to correct any miscalculation involved in the anticipatory control action inherent in feedforward (Goodwin et al., 2001).

The feedforward component $u_{ff}$ for a desired pressure is obtained from Eq. 14. The overall control law is

$$u=-k_{p}\tilde{x}\left(t\right)-k_{i}\int_{t_{0}}^{t}\tilde{x}\left(\tau \right)d\tau +u_{ff}$$ (36)

where $k_{p}$ and $k_{i}$ are the proportional and integral gains of the PI controller.

Considering the duty cycle of the valve is limited between 0 and 1 and large set point changes are usually desired in soft robotic applications, the integral term becomes unacceptably large leading to poor transient response (e.g., large settling time) without an anti-windup mechanism. Here, anti-windup is implemented using conditional integration (integrator clamping), where the integration is disabled when the duty cycle saturates (Visioli, 2006; Åström and Hägglund, 2006).

## 5 Simulation Results

### 5.1 Simscape Model

**FIGURE 2:**
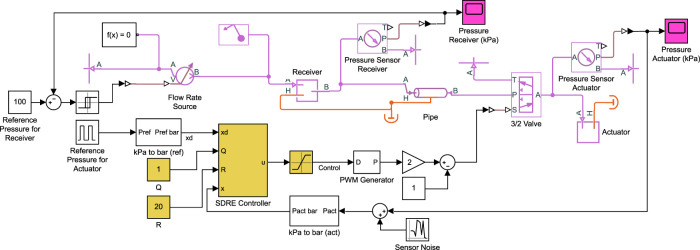
Model of pneumatic control system in Simulink. The diaphragm pump is modeled as a volumetric flow rate source, the pneumatic line as a pipe, and the air receiver and actuator as constant volume chambers. Here, the SDRE controller is shown in yellow, where the SDRE controller block contains the implementation of Eqs. 24 and 14. Equivalent blocks are used for the other controllers. Pressure sensors are added to the actuator and receiver and random sensor noise is added to the pressure measurements.

Using the mathematical model, the duty cycle is directly applied to the valve equations. However, in practical systems, the duty cycle is converted to a PWM wave and then applied to the valve. The system model is developed in Simscape Fluids within MATLAB/Simulink, as shown in Figure 2. The pneumatic components are shown in magenta and include the flow rate source, receiver, pipe, 3/2 solenoid valve, and the soft actuator. The actuator is modeled as a constant volume chamber. This is a reasonable assumption considering fiber-reinforced bending actuators, pneumatic network actuators, 3D/4D-printed actuators, and actuators fabricated with harder silicone rubbers exhibit low levels of volume change due to ballooning (Chen et al., 2020; Xavier et al., 2021b). Pressure sensors are added to the actuator and receiver, and random sensor noise with zero mean and 0.5-kPa variance is added to the pressure measurements.

### 5.2 Performance Evaluation

**FIGURE 3:**
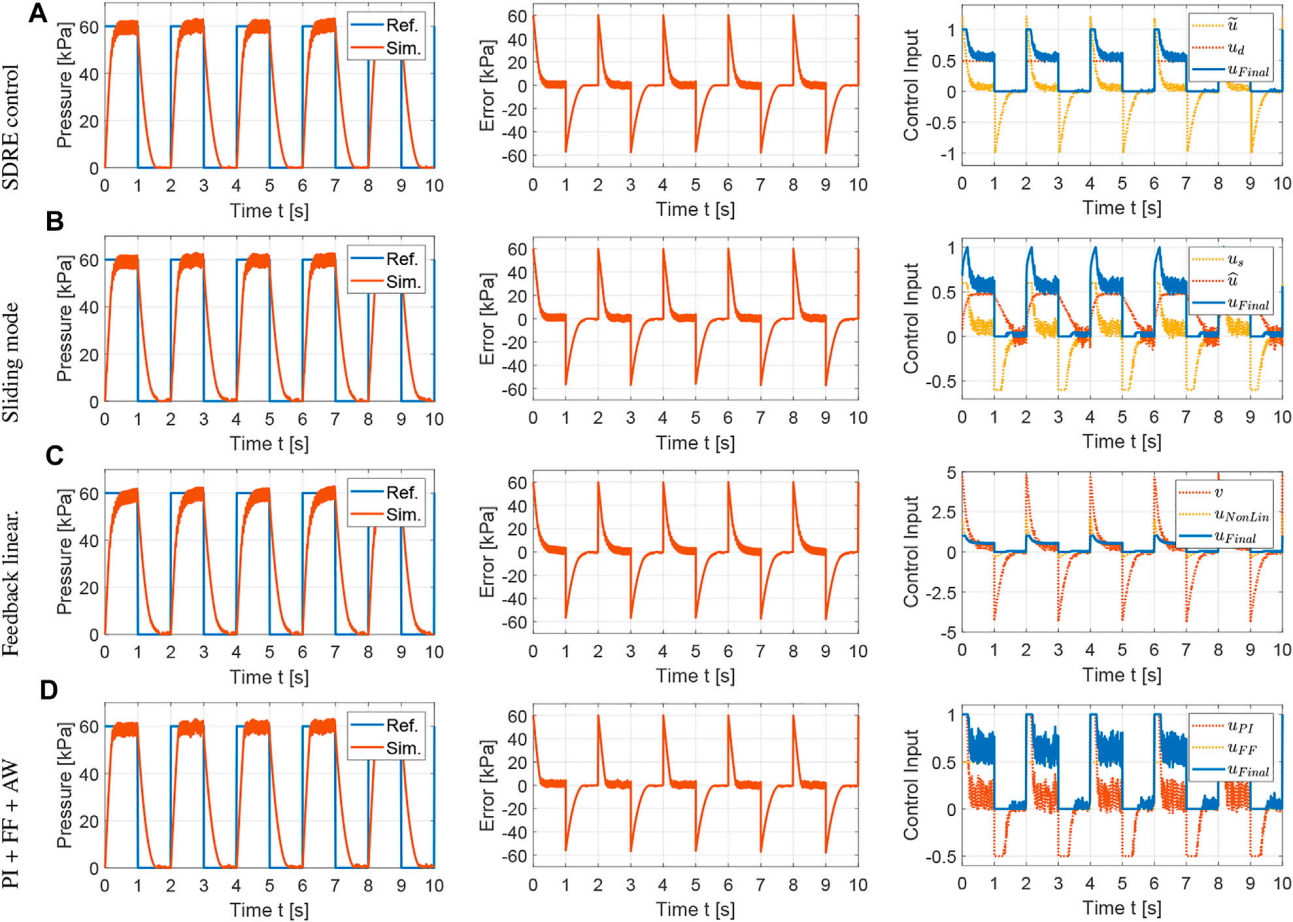
Simulation results for the comparison of nonlinear control strategies using the Simscape Fluids model: tracking performance, tracking error and control input for square wave with T = 2 s. **(A)** SDRE control: Q=5 and R=10 , $\tilde{u}$ is the feedback term, $u_{d}$ is the feedforward term, and $u_{Final}$ is the control applied to the valve after saturation. **(B)** Integral augmented sliding mode control: $k_{s}=0.6$ , $k_{i}=0.01$ and Φ=0.2 , $u_{s}$ is the discontinuous term, $\hat{u}$ is the equivalent control, and $u_{Final}$ is the control applied to the valve after saturation. **(C)** Feedback linearization with integral action: k=8 and $k_{i}=0.01$ , v is the linear control term, $u_{NonLin}$ is the total control, and $u_{Final}$ is the control applied to the valve after saturation. **(D)** PI controller with feedforward and anti-windup: $k_{p}=5$ and $k_{i}=0.5$ , $u_{PI}$ is the PI control term, $u_{FF}$ is the feedforward term, and $u_{Final}$ is the control applied to the valve after saturation.

The control methods proposed in Section 4 are evaluated with simulations using Simscape Fluids. A polytropic index of 1.2 is used in the controller design as this value showed excellent agreement between the mathematical and Simscape models. The tracking performance and control inputs for a reference square wave with a period of 2 s are shown in Figure 3. The controller parameters are tuned empirically *via* simulation to minimize the settling time and overshoot of the pressure responses following the general guidelines for each controller provided in Section 4.

The performance of these control strategies is evaluated by three metrics: average tracking error $\bar{e}$ , average control input $\bar{u}$ , and average control variation $\bar{\Delta u}$ , as given below,

$$\bar{e}=\frac{1}{N}\overset{N}{\underset{k=1}{\sum }}\left|e\left(k\right)\right|,$$ (37)

$$\bar{u}=\frac{1}{N}\overset{N}{\underset{k=1}{\sum }}\left|u\left(k\right)\right|,$$ (38)

$$\bar{\Delta u}=\frac{1}{N}\overset{N}{\underset{k=1}{\sum }}\left|u\left(k\right)-u\left(k-1\right)\right|.$$ (39)

**FIGURE 4:**
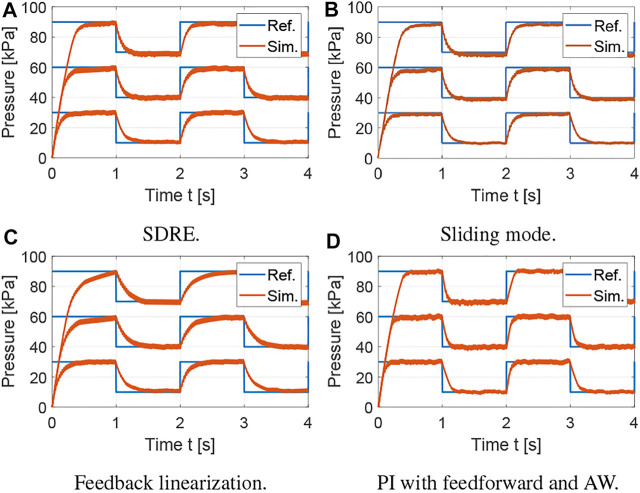
Evaluation of robustness of control strategies to variation in pressure references with varying offsets. **(A)** SDRE. **(B)** Sliding mode. **(C)** Feedback linearization. **(D)** PI with feedforward and AW.

**FIGURE 5:**
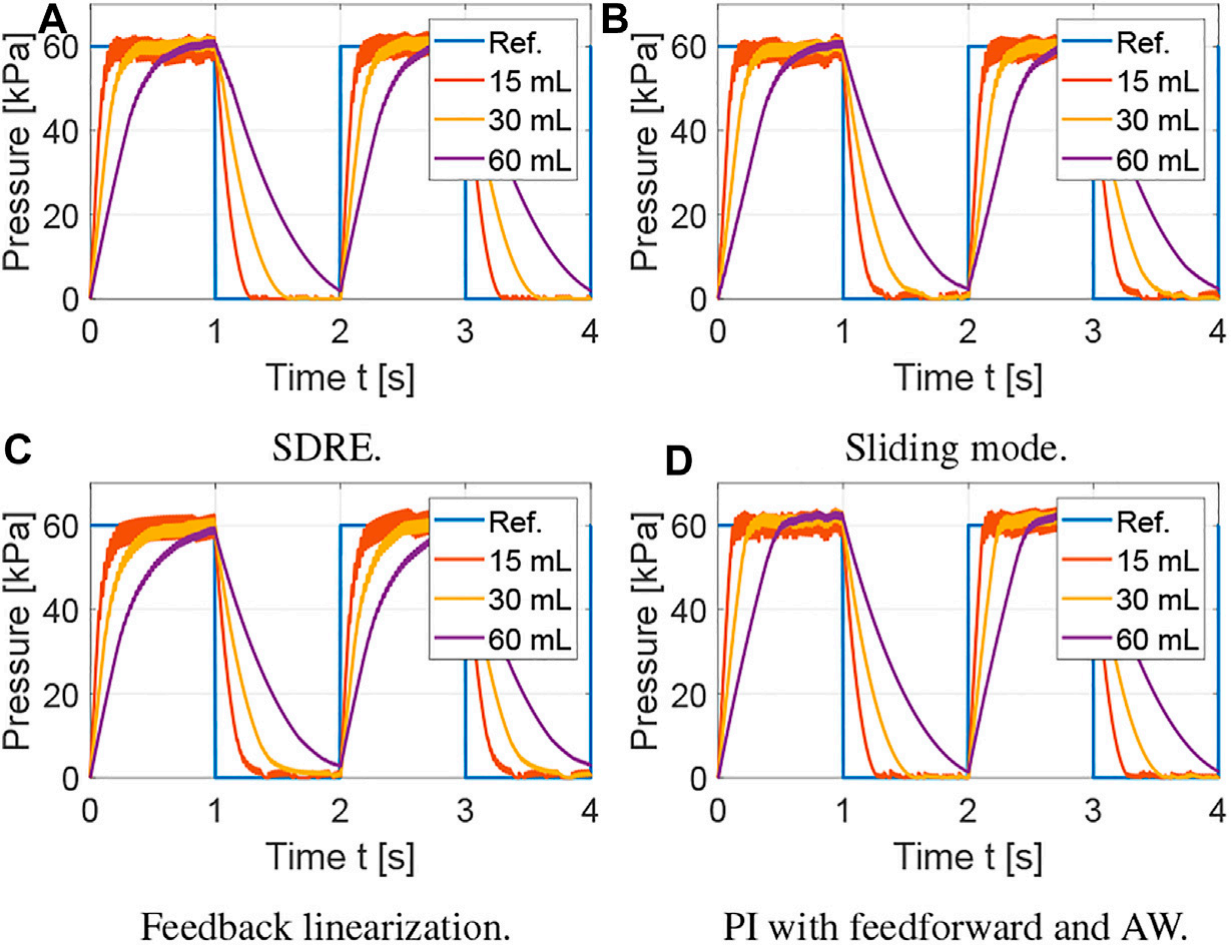
Evaluation of robustness of control strategies to variation in actuator volume. For each controller, the results are shown for three actuator volumes: 15, 30, and 60 ml. **(A)** SDRE. **(B)** Sliding mode. **(C)** Feedback linearization. **(D)** PI with feedforward and AW.

The average tracking error is used to evaluate the tracking performance, the average control input is used to evaluate the amount of control effort, and the average control variation is used to measure the degree of control input chattering. The metrics for the results shown in Figure 3 are summarized in Table 1 using the average over the five actuation cycles. Note that there are small differences between each cycle due to sensor noise, small variations in the receiver pressure, and the viscoelasticity of the silicone rubber. However, these differences are not significant, which supports the robustness of the proposed controllers. The SDRE and sliding mode controllers have similar tracking performance but the SDRE controller displays less chattering and control effort, and slightly faster settling time. Using feedback linearization, high proportional gains were needed to achieve tracking performance comparable to the previous optimal and robust nonlinear control strategies. Although feedback linearization resulted in less chattering, the tracking performance is inferior. Finally, it can be observed that, when the nonlinear model is incorporated into the feedforward component for a PI controller with anti-windup, excellent tracking performance can be achieved. To evaluate the robustness of the control strategies, a range of set points and soft actuator volumes are evaluated in Figures 4, 5, respectively. As shown in Figure 4, all controllers are able to track references at varying pressure ranges, except for the feedback linearization control law, which shows sluggish response for higher pressures. In addition, all controllers are robust to volume changes in the soft actuator, especially the SDRE, sliding mode, and PI with feedforward and anti-windup controllers. Although the actuator has some level of ballooning during actuation, the performance of the controllers is not significantly affected by the constant volume assumption.

**TABLE 1:** Performance of control strategies: simulation results for square wave with T = 2 s.

Control	$\bar{e}$	$\bar{u}$	$\bar{\Delta u}$
SDRE	9.0161	0.3441	0.0013
Integral augmented sliding mode	8.9435	0.3531	0.0028
Feedback linearization with integral action	9.2971	0.3367	0.0011
PI + Feedforward + Anti-windup	8.6755	0.3759	0.0029

## 6 Experimental Results

**FIGURE 6:**
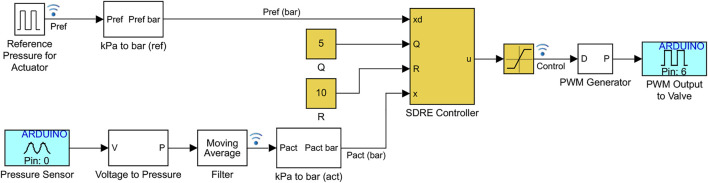
Implementation of control strategies with Simulink and Arduino. Here, the SDRE controller is shown in yellow, equivalent blocks are used for the other controllers. The blue blocks indicate the pressure sensor measurements (analog input) and PWM wave applied to the 3/2 valve (digital output).

The actuator used in the experimental results is a pneumatic network bending actuator fabricated using standard molding procedures (Marchese et al., 2015; Schmitt et al., 2018), as shown in Figure 1. Molds are designed in Autodesk Inventor and printed using an Original Prusa i3 MK3S (Prusa Research). Silicone rubber (DragonSkin10) forms the main body of the actuator and a strain limiting layer of fiberglass fabric is added to the bottom of the actuator to generate bending. The control strategies are programmed using Simulink; then, the Simulink support package for Arduino hardware and the MinGW64 compiler are used to generate code and interactively communicate in real time with an Arduino Due at a sampling time of 5 ms. To allow for real-time control and data visualization, Simulink is run in external mode over the serial and desired signals are logged for analysis with the Simulation Data Inspector. To reduce the noise level in the pressure sensor, the pressure data is filtered using a moving average filter for the last 10 pressure measurements. An example of the Simulink-Arduino implementation is shown in Figure 6 for the SDRE controller.

### 6.1 Performance Evaluation and Comparison to Simulations

**FIGURE 7:**
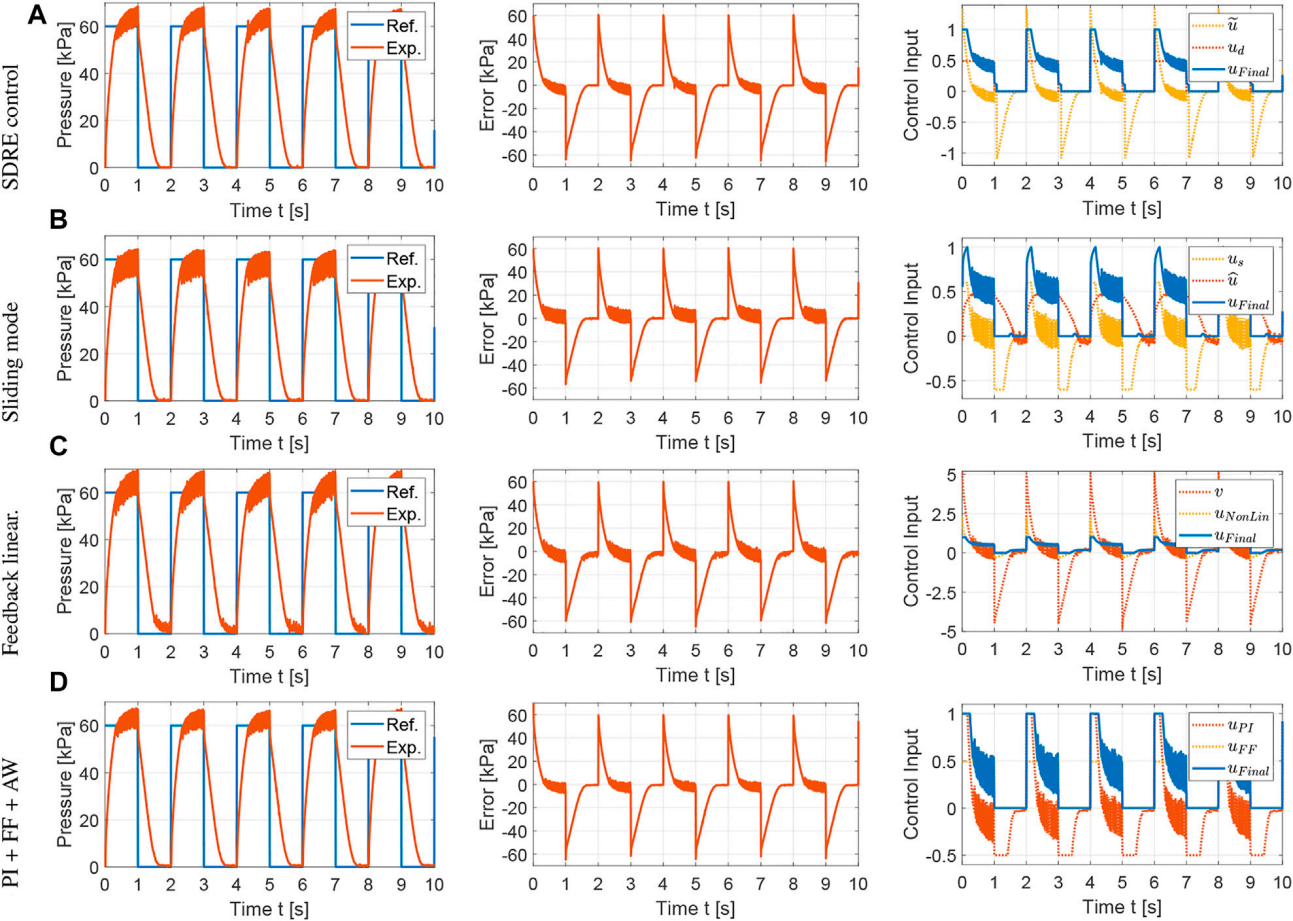
Experimental results for the comparison of nonlinear control strategies: tracking performance, tracking error and control input for square wave with T = 2 s. **(A)** SDRE control: Q=5 and R=10 . **(B)** Integral augmented sliding mode control: $k_{s}=0.6$ , $k_{i}=0.01$ , and Φ=0.2 . **(C)** Feedback linearization with integral action: k=8 and $k_{i}=0.01$ . **(D)** PI controller with feedforward and anti-windup: $k_{p}=5$ and $k_{i}=0.5$ . The description for the control input terms is the same as in Figure 3.

The tracking performance and control inputs for a reference square wave with a period of 2 s are shown in Figure 7. Compared to the simulations, the experimental results show an increased level of chattering due to sensor noise and compliance of the soft actuator. The SDRE and integral augmented sliding mode controllers provided the best tracking performances, with the SDRE controller showing reduced chattering and control effort, as summarized in Table 2. Comparing Tables 1 and 2, it is clear that the PI controller augmented with feedforward and anti-windup performs better in simulation results. However, its performance is still comparable to nonlinear optimal and robust control strategies, i.e., SDRE and SMC, respectively. The rise time for all controller responses are below 0.5 s, which validates the open-loop design procedure for the pneumatic components in Section 3. The response time can be further reduced with larger receiver pressures or valve flow coefficients. It is important to note that the response time is also a function of the internal chamber design of the soft actuator and the length and diameter of the tube connection, which can effectively act as flow restrictions. In this work, a tube with internal diameter of 4 mm and length of 8 cm between the valve and the soft actuator was used to minimize flow resistance and the added volume in the flow path.

**TABLE 2:** Performance of control strategies: experimental results for square wave with T = 2 s.

Control	$\bar{e}$	$\bar{u}$	$\bar{\Delta u}$
SDRE	12.1286	0.2982	0.0261
Integral augmented sliding mode	11.2302	0.3068	0.0423
Feedback linearization with integral action	13.3625	0.3595	0.0198
PI + Feedforward + Anti-windup	12.6215	0.3013	0.0485

**FIGURE 8:**
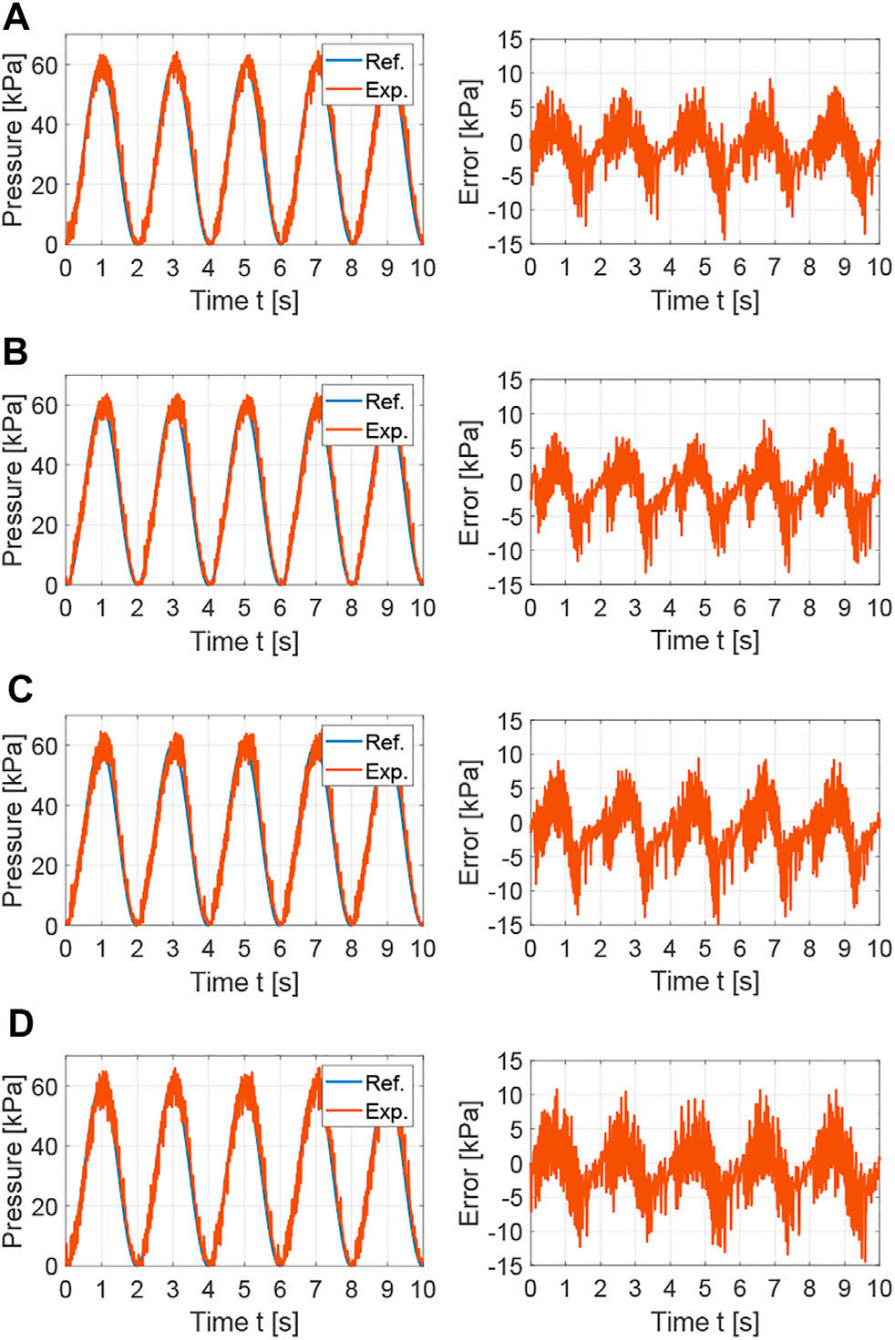
Experimental results for the comparison of nonlinear control strategies: tracking performance and error for sine wave with T = 2 s. **(A)** SDRE control: Q=50 and R=1 . **(B)** Integral augmented sliding mode control: $k_{s}=2$ , $k_{i}=0.01$ and Φ=0.2 . **(C)** Feedback linearization with integral action: k=40 and $k_{i}=0.01$ . **(D)** PI controller with feedforward and anti-windup: $k_{p}=10$ and $k_{i}=0.5$ .

The tracking performance of the nonlinear controllers is also evaluated for a sine wave with a period of 2 s, as shown in Figure 8. As for the square waves, the SDRE and integral augmented sliding mode controllers provided the best tracking performances with average tracking errors of 2.694 and 2.729, respectively. The feedback linearization controller with integral action and the PI controller augmented with feedforward and anti-windup showed average tracking errors of 3.942 and 3.139.

The chattering observed in the actuator responses in Figures 7, 8 is an inherent characteristic of pneumatic systems with on/off 3/2 valves, where flow is released to atmosphere at the off state of the PWM wave during pressure regulation, which causes the pressure in the actuator to continuously oscillate around its target. This can be reduced using systems with dual on/off 2/2 valves, which allow for an intermediate state where there is no flow in or out of the soft actuator. However, these systems increase control complexity as two inputs are controlled and cost as two valves are required. Alternatively, proportional valves can be used. However, these valves generally are more costly and much larger in size than on/off 3/2 or 2/2 solenoid valves.

## 7 Conclusion

This article describes the application and comparison of model-based nonlinear feedback controllers for soft pneumatic actuators operated with on/off valves. A model of the pneumatic system is developed, and four control strategies are compared using simulation and experimental results. The SDRE and integral augmented sliding mode controllers exhibited excellent tracking performance in both simulations and experiments. However, the SDRE controller showed less chattering and control effort compared to the sliding mode controller. The PI controller with feedforward and anti-windup performs better in simulations.

Integral action was introduced to the sliding mode and feedback linearization control laws to provide some robustness to model uncertainties. The three most uncertain parameters were as follows: the flow coefficient of the valve, the receiver pressure which decreases slightly during charging, and the actuator volume which increases with pressure. All four controllers showed robustness to varying actuator pressures and volumes. However, the feedback linearization method was noticeably slower at higher pressures and volumes.

On the basis of the experimental results, the authors recommend the SDRE method due to the systematic design process and best combination of fast response and minimum chatter. Whereas some nonlinear control strategies only address stability, the SDRE method directly addresses performance through the specification of a performance index in the nonlinear regulator problem. Furthermore, the state and control weightings can be adjusted with predictable results similar to linear quadratic regulator designs.

## Data Availability

The original contributions presented in the study are included in the article, further inquiries can be directed to the corresponding author.

## Appendix-Error System for SDRE Control

Firstly, define the reference system

$$\begin{array}{cc} {\dot{x}}_{d}= & -\alpha x_{d}\underset{a_{1d}}{\underset{︸}{\sqrt{c_{2}\left(x_{d}-c_{2}\right)}}}\\ & +\left(\alpha x_{d}\underset{a_{2d}}{\underset{︸}{\sqrt{x_{d}\left(c_{1}-x_{d}\right)}}}+\alpha x_{d}\underset{a_{1d}}{\underset{︸}{\sqrt{c_{2}\left(x_{d}-c_{2}\right)}}}\right)u_{d} \end{array}$$
(40)

From the definition of the error variables, $x=x_{d}+\tilde{x}$ and $u=u_{d}+\tilde{u}$ . Therefore, in Eqs. 8–10,

$$\begin{array}{cc} {\dot{x}}_{d}+\dot{\tilde{x}}= & -\alpha \left(x_{d}+\tilde{x}\right)\underset{a_{1}}{\underset{︸}{\sqrt{c_{2}\left(x_{d}+\tilde{x}-c_{2}\right)}}}\\ & +\left\{\alpha \left(x_{d}+\tilde{x}\right)\right.\underset{a_{2}}{\underset{︸}{\sqrt{\left(x_{d}+\tilde{x}\right)\left(c_{1}-x_{d}-\tilde{x}\right)}}}\\ & +\alpha \left(x_{d}+\tilde{x}\right)\underset{a_{1}}{\underset{︸}{\sqrt{c_{2}\left(x_{d}+\tilde{x}-c_{2}\right)}}}\}\left(u_{d}+\tilde{u}\right) \end{array}$$
(41)

Using the reference system (Eq. 40) and the definitions of $a_{1}$ , $a_{2}$ , $a_{1d}$ , and $a_{2d}$ ,

$$\begin{array}{cc} \dot{\tilde{x}}= & -\alpha \tilde{x}a_{1}+\alpha \tilde{x}a_{2}u_{d}+\alpha \tilde{x}a_{1}u_{d}\\ & +\left(\alpha x_{d}a_{2}+\alpha \tilde{x}a_{2}+\alpha x_{d}a_{1}+\alpha \tilde{x}a_{1}\right)\tilde{u}\\ & -\alpha x_{d}a_{1}+\alpha x_{d}a_{2}u_{d}+\alpha x_{d}a_{1}u_{d}\\ & +\alpha x_{d}a_{1d}-\alpha x_{d}a_{2d}u_{d}-\alpha x_{d}a_{1d}u_{d} \end{array}$$
(42)

The last two lines in the equation above define $a_{3}$ , multiplying $a_{3}$ by $\tilde{x}/\tilde{x}$ yields

$$\begin{array}{cc} \dot{\tilde{x}}= & \underset{A\left(\tilde{x}\right)}{\underset{︸}{\left(-\alpha a_{1}+\alpha a_{2}u_{d}+\alpha a_{1}u_{d}+a_{3}/\tilde{x}\right)}}\tilde{x}\\ & +\underset{B\left(\tilde{x}\right)}{\underset{︸}{\left(\alpha x_{d}a_{2}+\alpha \tilde{x}a_{2}+\alpha x_{d}a_{1}+\alpha \tilde{x}a_{1}\right)}}\tilde{u} \end{array}$$
(43)
